# 99 Delivery of advice on physical activity by general practitioners in Germany: a cross-sectional population survey in people with chronic ischemic heart disease (OptiCor study)

**DOI:** 10.1093/eurpub/ckae114.056

**Published:** 2024-09-26

**Authors:** Sabrina Hoppe, Daniel Kotz, Ute Mons, Alicia Prinz, Sabrina Kastaun

**Affiliations:** Institute of General Practice (ifam), Patient-Physician-Communication Research Unit, Centre for Health and Society (chs), Medical Faculty and University Hospital Düsseldorf, Heinrich-Heine-University, Düsseldorf, Germany; Institute of General Practice (ifam), Addiction Research and Clinical Epidemiology Unit, Centre for Health and Society (chs), Medical Faculty and University Hospital Düsseldorf, Heinrich-Heine-University, Düsseldorf, Germany; Department of Family Medicine, Care and Public Health Research Institute (CAPHRI), Maastricht University, Maastricht, The Netherlands; Department of Cardiology, Faculty of Medicine and University Hospital Cologne, University of Cologne, Cologne, Germany; Institute of General Practice (ifam), Patient-Physician-Communication Research Unit, Centre for Health and Society (chs), Medical Faculty and University Hospital Düsseldorf, Heinrich-Heine-University, Düsseldorf, Germany; Institute of General Practice (ifam), Patient-Physician-Communication Research Unit, Centre for Health and Society (chs), Medical Faculty and University Hospital Düsseldorf, Heinrich-Heine-University, Düsseldorf, Germany

## Abstract

**Purpose:**

The current German treatment guideline “chronic ischemic heart disease (IHD)” recommends that general practitioners (GPs) deliver advice on physical activity (PA) to patients with IHD. Such advice is particularly effective in motivating individuals to increase their PA when it consists of three elements: (1) asking for/assessing the PA level, (2) advising on PA, and (3) assisting with PA (e.g., providing concrete support/recommendations). However, in Germany, current and representative data on the receipt of these elements of GP advice in individuals with IHD is lacking. This study aims at collecting such data on a population level.

**Methods:**

Nationwide, computer-assisted, face-to-face cross-sectional survey of ≃1000 individuals (35+ years) with self-reported IHD on the receipt of the three elements of GP advice on PA (ask/assess, advise, assist) since their last remembered IHD-event/intervention (e.g., myocardial infarction, stent insertion). The survey started in June 2023 and will continue until May 2024. Six of eight survey waves have been completed. When the complete data set is available, associations with person characteristics (e.g., sex, income, region of living, body mass index, PA level) will be analysed using adjusted regression models. Results on the associations will be presented at the conference.

**Results:**

Of the current sample of 682 individuals with IHD, 35.3% (95% confidence interval (CI) = 32-39) received all three elements of GP advice (ask/assess, advise, assist), 44.4% (95%CI = 41-48) received one or two elements (e.g., ask/assess only), and 10.0% (95%CI = 8-12) received any advice (2.9% received the advice to refrain from PA, 7.3% did not remember/refused to answer). Men (38.0%; 95%CI = 34-43) versus women (30.5%; 95%CI = 25-36), individuals from urban (40.7%; 95%CI = 36-46) versus rural regions (26.2%; 95%CI = 21-32), and those with a PA level of 1-149 minutes/week (43.1%; 95%CI = 36-51) and of 150+ minutes/week (39.0%; 95%CI = 31-47) versus non-active individuals (20.7%; 95%CI = 15-28) reported the receipt of all elements of PA advice more often.

**Conclusions:**

In Germany, about one third of individuals with IHD received all relevant elements of GP advice on PA since their last IHD-event/intervention. In order to increase the delivery and enhance the effectiveness of GP advice, target group-specific measures need to be implemented.

**Funding:**

German Ministry of Education and Research (01GY2103).

